# Supplementary material for: Differential Drought Responses of Soybean Genotypes in Relation to Photosynthesis and Growth-Yield Attributes
Source: Plants (Basel). 2024 Oct 2;13(19):2765. doi: 10.3390/plants13192765 (PMC11478663; doi:10.3390/plants13192765)
Supplement: Supplementary file 1 [file plants-13-02765-s001.zip › plants-3171098-supplementary.docx]

**Supplemental Table S1.** Analysis of variances (ANOVA) of studied traits under varying water regime conditions

| **Studied traits** | **Mean ± Standard Error of Mean** | | **Mean sum of squares** | | **Coefficient of variations (%)** | | ***F*-value** | | ***p*-value** | |
| --- | --- | --- | --- | --- | --- | --- | --- | --- | --- | --- |
|  | **control** | **drought** | **control** | **drought** | **control** | **drought** | **control** | **drought** | **control** | **drought** |
| Plant height | 52.21±1.08 | 36.93±1.12 | 180.65 | 139.13 | 2.95 | 3.63 | 38.417 | 27.77 | 2.92E-10^***^ | 5.99E-09^***^ |
| Leaf area plant^–1^ | 574.65±29.70 | 342.92±17.59 | 62761.00 | 21134.50 | 10.36 | 9.79 | 17.794 | 17.08 | 3.03E-07^***^ | 4.28E-07^***^ |
| Leaves number plant^–1^ | 22.30±0.83 | 15.78±0.47 | 112.24 | 25.21 | 6.69 | 6.22 | 40.741 | 28.65 | 1.67E-10^***^ | 4.49E-09^***^ |
| Branches number plant^–1^ | 3.51±0.15 | 2.24±0.12 | 2.07 | 0.80 | 7.87 | 10.85 | 23.056 | 14.43 | 3.19E-08^***^ | 1.73E-06^***^ |
| Pods number plant^–1^ | 20.41±0.72 | 10.75±0.46 | 201.25 | 179.22 | 6.83 | 8.73 | 96.183 | 211.94 | 3.59E-14^***^ | <2.20E-16^***^ |
| Seeds pod^–1^ | 2.16±0.09 | 1.48±0.05 | 0.31 | 0.66 | 9.12 | 6.06 | 8.9661 | 68.09 | 6.42E-05^***^ | 1.13E-12^***^ |
| 100-seed weight | 16.16±0.53 | 10.64±0.46 | 39.66 | 31.51 | 6.34 | 7.07 | 35.736 | 37.21 | 5.78E-10^***^ | 3.95E-10^***^ |
| Seed yield plant^–1^ | 6.28±0.27 | 3.11±0.12 | 25.13 | 22.73 | 7.16 | 7.83 | 88.343 | 413.14 | 8.44E-14^***^ | <2.20E-16^***^ |
| Relative water content | 87.73±1.45 | 66.54±2.92 | 22.22 | 155.22 | 2.78 | 7.8 | 2.64 | 4.56 | 0.045^*^ | 0.004^**^ |
| Water saturation deficit | 11.82±1.23 | 33.46±1.11 | 6.65 | 155.22 | 20.25 | 5.66 | 1.09 | 31.22 | 0.395 | 2.04E-09^***^ |
| Water retention capacity | 5.63±0.24 | 5.58±0.22 | 0.44 | 0.47 | 8.37 | 7.41 | 1.90 | 2.43 | 0.1281 | 0.06063 |
| Water uptake capacity | 0.55±0.04 | 1.56±0.12 | 0.02 | 0.49 | 15.48 | 14.47 | 2.79 | 9.23 | 0.03688^*^ | 5.24E-05^***^ |
| Xylem exudation rate | 0.39±0.02 | 0.18±0.01 | 0.02 | 0.01 | 9.38 | 8.31 | 13.9 | 42.25 | 2.30E-06^***^ | 1.18E-10^***^ |
| Stomatal conductance | 0.25±0.01 | 0.07±0.003 | 0.06 | 0.01 | 7.79 | 9.74 | 153.25 | 268.28 | 3.18E-16^***^ | <2.20E-16^***^ |
| Photosynthetic rate | 14.83±0.44 | 8.02±0.18 | 68.70 | 65.64 | 4.65 | 4.37 | 87.33 | 490.40 | 9.48E-14^***^ | <2.20E-16^***^ |
| Inter. CO_2_ concentration | 316.85±3.26 | 347.26±3.52 | 18979.30 | 21433.10 | 1.85 | 2.13 | 445.50 | 432.08 | <2.20E-16^***^ | <2.20E-16^***^ |
| Transpiration rate | 5.25±0.13 | 3.23±0.14 | 17.08 | 15.32 | 3.47 | 7.93 | 265.95 | 204.44 | <2.20E-16^***^ | <2.20E-16^***^ |
| Leaf temperature | 30.02±0.18 | 32.36±0.14 | 16.93 | 31.04 | 1.15 | 0.86 | 131.05 | 418.16 | 1.57E-15^***^ | <2.20E-16^***^ |
| Int. water use efficiency | 65.94±4.16 | 157.04±11.72 | 1110.49 | 18793.90 | 13.1 | 13.67 | 16.011 | 34.19 | 7.35E-07^***^ | 8.76E-10^***^ |
| Ins. water use efficiency | 2.97±0.14 | 2.67±0.15 | 0.81 | 1.19 | 8.31 | 11.15 | 10.70 | 13.18 | 1.78E-05^***^ | 3.56E-06^***^ |
| Chlorophyll *a* | 3.62±0.12 | 1.90±0.08 | 2.45 | 1.77 | 7.27 | 9.46 | 40.02 | 62.04 | 1.98E-10^***^ | 2.84E-12^***^ |
| Chlorophyll *b* | 1.94±0.10 | 1.11±0.05 | 0.80 | 0.12 | 10.28 | 8.79 | 21.26 | 13.00 | 6.53E-08^***^ | 3.97E-06^***^ |
| Total Chlorophylls | 5.55±0.13 | 3.01±0.06 | 5.42 | 2.24 | 4.62 | 4.09 | 78.19 | 158.37 | 2.86E-13^***^ | 2.27E-16^***^ |
| Carotenoids | 8.28±0.24 | 5.34±0.10 | 1.36 | 5.50 | 5.85 | 4.12 | 6.11 | 125.37 | 0.00079^***^ | 2.46E-15^***^ |
| Proline | 2.40±0.12 | 7.79±0.14 | 3.31 | 25.71 | 9.94 | 3.20 | 53.60 | 342.26 | 1.19E-11^***^ | <2.20E-16^***^ |
| Malondialdehyde | 3.69±0.17 | 9.39±0.33 | 4.77 | 130.00 | 8.03 | 6.92 | 39.11 | 307.71 | 2.47E-10^***^ | <2.20E-16^***^ |

Int., intrinsic; Ins., instantaneous; Inter., intercellular. Statistical analyses were conducted separately for drought and control treatments, with values derived from five biological replicates (*n* = 5; 10 plants per replicate). Asterisks designate significance levels where * *p* ≤ 0.05, ** *p* ≤ 0.01 and *** *p* ≤ 0.01.

**Supplemental Table S2.** Morphological traits of investigated soybean genotypes under varying water regime conditions

| **Genotypes** | **Plant height**  **(cm)** | | **Leaf area plant^–1^ (cm^2^)** | | **Leaves number plant^–1^** | | **Branches number plant^–1^** | | **Pods number plant^–1^** | | **Seeds pod^–1^** | | | | **100-seed weight (g)** | | | **Seed yield plant^–1^ (g)** | | |
| --- | --- | --- | --- | --- | --- | --- | --- | --- | --- | --- | --- | --- | --- | --- | --- | --- | --- | --- | --- | --- |
|  | **control** | **drought** | **control** | **drought** | **control** | **drought** | **control** | **drought** | **control** | **drought** | | **control** | **drought** | **control** | | **drought** | **control** | | **drought** |  |
| G00001 | 44.5^e^ | 36.6^d^  (17.6) | 761.1^a^ | 483.7^a^  (36.4) | 17.9^c^ | 13.6^d^  (24.5) | 3.1^c^ | 2.2^b^  (27.3) | 17.6^c^ | 15.5^b^  (12.2) | 2.5^a^ | | 1.9^a^  (22.0) | 21.4^a^ | | 14.6^a^  (31.6) | 7.7^b^ | | 5.8^b^  (24.6) |  |
| G00046 | 57.3^b^ | 45.6^a^  (20.4) | 457.6^c^ | 366.1^b^  (20.0) | 27.2^a^ | 18.3^ab^  (33.0) | 4.0^ab^ | 2.8^a^  (31.3) | 31.8^a^ | 20.8^a^  (34.6) | 2.1^b^ | | 1.7^b^  (20.0) | 16.1^c^ | | 13.2^b^  (18.0) | 10.1^a^ | | 6.6^a^  (34.6) |  |
| G00135 | 44.4^e^ | 28.2^f^  (36.6) | 632.8^b^ | 303.3^c^  (52.1) | 15.1^d^ | 13.3^d^  (11.8) | 2.4^d^ | 1.9^cd^  (25.3) | 24.5^b^ | 12.1^c^  (50.8) | 2.3^ab^ | | 1.4^c^  (38.5) | 13.4^e^ | | 8.4^d^  (37.6) | 4.7^d^ | | 1.5^d^  (67.8) |  |
| BD2333 | 53.0c | 33.4^e^  (37.1) | 627.9^b^ | 369.9^b^  (41.1) | 26.8^a^ | 17.2^b^  (35.7) | 4.3^a^ | 1.7^d^  (61.9) | 15.2^d^ | 2.2^e^  (85.9) | 1.8^c^ | | 0.8^e^  (56.2) | 13.5^e^ | | 6.2e  (54.2) | 3.3^e^ | | 1.1^e^  (67) |  |
| PK472 | 63.1^a^ | 42.4^b^  (32.8) | 417.2^c^ | 308.5^c^  (26.1) | 23.7^b^ | 15.0^c^  (36.6) | 3.7^b^ | 2.6^a^  (29.5) | 24.3^b^ | 12.9^c^  (46.7) | 2.4^a^ | | 1.7^b^  (29.9) | 13.9^de^ | | 10.7^c^  (23.0) | 7.6^bc^ | | 3.3^c^  (56.8) |  |
| BARI Soybean6 | 52.7^c^ | 33.6^e^  (36.2) | 647.8^b^ | 306.9^c^  (52.6) | 27.7^a^ | 19.5^a^  (29.7) | 4.4^a^ | 2.7^a^  (36.1) | 10.2^e^ | 2.8^e^  (72.7) | 1.7^c^ | | 1.1^d^  (37.1) | 15.3^cd^ | | 11.0c  (27.7) | 3.5^e^ | | 1.4^d^  (60.7) |  |
| BU Soybean2 | 50.4^d^ | 38.9^c^  (22.9) | 478.2^c^ | 261.9^c^  (45.2) | 17.7^c^ | 13.8^cd^  (21.7) | 3.0^c^ | 2.1^bc^  (30.0) | 19.2^c^ | 9.2^d^  (52.2) | 2.3^ab^ | | 1.8^b^  (22.2) | 19.6^b^ | | 10.6c  (46.0) | 7.1^c^ | | 3.1^c^  (55.6) |  |
| LSD _0.05_ | 2.28 | 1.99 | 88.4 | 49.9 | 2.21 | 1.46 | 0.4 | 0.36 | 2.07 | 1.40 | 0.29 | | 0.13 | 1.52 | | 1.12 | 0.67 | | 0.36 |  |
| CV (%) | 2.95 | 3.63 | 10.36 | 9.79 | 6.69 | 6.22 | 7.87 | 10.85 | 6.83 | 8.73 | 9.12 | | 6.06 | 6.34 | | 7.07 | 7.16 | | 7.83 |  |

LSD, least significant difference; CV, coefficient of variations. Statistical analyses were conducted separately for drought and control treatments, with values derived from five biological replicates (*n* = 5; 10 plants per replicate). Distinct alphabetical symbols denote the significant differences (*p* < 0.05) of genotypes for the traits analyzed, with a > b > c > d > e. Value in the parenthesis indicates the percent reduction under drought stress over control.

**Supplemental Table S3.** Photosynthetic features of investigated soybean genotypes under varying water regime conditions

| **Genotypes** | **Stomatal conductance**  **(mol H_2_O m^–2^ s^–1^)** | | **Photosynthetic rate**  **(μmol CO_2_ m^–2^ s^–1^)** | | **Intercellular CO_2_ concentration* (ppm)** | | **Transpiration rate (mmol H_2_O m^–2^ s^–1^)** | | **Leaf temperature* (^o^C)** | | **Intrinsic water use efficiency***  **(μmol CO_2_ mol^–1^ H_2_O)** | | **Instantaneous water use efficiency (μmol CO_2_ mol^–1^ H_2_O)** | |
| --- | --- | --- | --- | --- | --- | --- | --- | --- | --- | --- | --- | --- | --- | --- |
|  | **control** | **drought** | **control** | **drought** | **control** | **drought** | **control** | **drought** | **control** | **drought** | **control** | **drought** | **control** | **drought** |
| G00001 | 0.45^a^ | 0.15^a^  (66.8) | 19.60^a^ | 13.73^a^  (30.0) | 254.77^f^ | 291.30^e^  (14.3) | 8.29^a^ | 6.19^a^  (25.3) | 26.61^f^ | 27.48^f^  (3.3) | 43.48^c^ | 95.02^e^  (118.5) | 2.37^e^ | 2.22^d^  (6.3) |
| G00046 | 0.35^b^ | 0.09^b^  (73.3) | 20.01^a^ | 12.18^b^  (39.1) | 272.60^e^ | 288.39^e^  (5.8) | 7.14^b^ | 5.12^b^  (28.3) | 28.42^e^ | 30.53^e^  (7.4) | 57.93^b^ | 128.96^cd^  (122.6) | 2.80^b-d^ | 2.40^cd^  (14.6) |
| G00135 | 0.12^f^ | 0.01^e^  (89.2) | 10.30^d^ | 4.44^e^  (56.9) | 394.49^b^ | 431.53^b^  (9.4) | 2.75^g^ | 1.19^e^  (56.8) | 31.38^b^ | 34.62^b^  (10.3) | 89.47^a^ | 288.54^a^  (222.5) | 3.79^a^ | 3.78^a^  (0.3) |
| BD2333 | 0.16^e^ | 0.02^e^  (90.5) | 10.18^d^ | 2.95^f^  (71.0) | 411.53^a^ | 445.28^a^  (8.2) | 3.22^f^ | 1.31^e^  (59.4) | 32.85^a^ | 35.60^a^  (8.4) | 65.17^b^ | 208.48^b^  (219.9) | 3.17^b^ | 2.28^d^  (28.0) |
| PK472 | 0.16^e^ | 0.06^c^  (66.8) | 13.45^c^ | 6.24^d^  (53.6) | 317.85^d^ | 344.90^d^  (8.5) | 4.32^e^ | 2.30^d^  (46.8) | 31.10^b^ | 33.47^c^  (7.6) | 87.13^a^ | 110.91^de^  (27.3) | 3.11^bc^ | 2.73^bc^  (12.5) |
| BARI Soybean6 | 0.22^d^ | 0.04^d^  (81.1) | 13.01^c^ | 6.46^d^  (50.3) | 335.41^c^ | 372.69^c^  (11.1) | 4.64^d^ | 2.25^d^  (51.4) | 30.17^c^ | 33.54^c^  (11.2) | 58.05^b^ | 147.86^c^  (154.7) | 2.80^cd^ | 2.79^b^  (0.4) |
| BU Soybean2 | 0.29^c^ | 0.09^b^  (70.4) | 17.30^b^ | 10.13^c^  (41.5) | 231.30^g^ | 256.75^f^  (11.0) | 6.35^c^ | 4.22^c^  (33.7) | 29.62^d^ | 31.27^d^  (5.6) | 60.33^b^ | 119.54^c-e^  (98.2) | 2.72^de^ | 2.41^cd^  (11.7) |
| LSD _(0.05)_ | 0.03 | 0.009 | 1.02 | 0.52 | 8.72 | 10.96 | 0.27 | 0.38 | 0.51 | 0.41 | 12.84 | 31.90 | 0.37 | 0.44 |
| CV (%) | 7.79 | 9.74 | 4.65 | 4.37 | 1.85 | 2.13 | 3.47 | 7.93 | 1.15 | 0.86 | 13.10 | 13.67 | 8.31 | 11.15 |

LSD, least significant difference; CV, coefficient of variations. Statistical analyses were conducted separately for drought and control treatments, with values derived from five biological replicates (*n* = 5; 10 plants per replicate). Value in the parenthesis indicates the percent reduction and increased under drought stress compared to control. Distinct alphabetical symbols denote the order of significance (p < 0.05) for the traits analyzed, with a > b > c > d > e > f > g. The parameters which have been marked with asterisk (*) that were increased under drought stress compared to control.

**Supplemental Table S4.** Plant water relations of investigated soybean genotypes under varying water regime conditions

| **Genotypes** | **Relative water content**  **(%)** | | **Water saturation deficit***  **(%)** | | **Water retention capacity** | | **Water uptake capacity*** | | **Xylem exudation rate**  **(g hour^–1^)** | |
| --- | --- | --- | --- | --- | --- | --- | --- | --- | --- | --- |
|  | **control** | **drought** | **control** | **drought** | **control** | **drought** | **control** | **drought** | **control** | **drought** |
| G00001 | 84.49^d^ | 70.59^ab^  (16.5) | 14.26^a^ | 29.41^e^  (106.3) | 5.07^c^ | 5.31^bc^  (4.7) | 0.67^a^ | 1.29^de^  (92.5) | 0.41^bc^ | 0.25^a^  (39.4) |
| G00046 | 86.69^b-d^ | 77.63^a^  (10.5) | 11.81^ab^ | 22.37^f^  (89.5) | 5.45^bc^ | 5.41^bc^  (0.7) | 0.54^bc^ | 0.99^e^  (84.6) | 0.50^a^ | 0.20^b^  (61.0) |
| G00135 | 88.12^a-d^ | 65.71^b-d^  (25.4) | 11.88^ab^ | 34.29^cd^  (188.6) | 5.68^a-c^ | 5.12^c^  (10.0) | 0.55^a-c^ | 1.48^cd^  (167.0) | 0.34^d^ | 0.10^d^  (71.3) |
| BD2333 | 91.21^a^ | 59.84^d^  (34.4) | 10.29^b^ | 40.16^a^  (290.4) | 6.15^a^ | 6.09^a^  (1.1) | 0.48^c^ | 2.02^a^  (324.2) | 0.45^ab^ | 0.19^b^  (57.2) |
| PK472 | 85.49^cd^ | 60.64^cd^  (29.1) | 12.26^ab^ | 39.36^ab^  (221.1) | 5.61^a-c^ | 5.70^a-c^  (1.6) | 0.61^ab^ | 1.87^ab^  (208.3) | 0.39^cd^ | 0.21^b^  (45.9) |
| BARI Soybean6 | 89.76^ab^ | 68.08^bc^  (24.2) | 10.62^b^ | 31.93^de^  (200.8) | 5.69^a-c^ | 5.90^ab^  (3.7) | 0.46^c^ | 1.55^b-d^  (237.5) | 0.38^cd^ | 0.12^c^  (68.2) |
| BU Soybean2 | 88.36^a-c^ | 63.27^b-d^  (28.4) | 11.65^ab^ | 36.73^bc^  (215.4) | 5.79^ab^ | 5.53^a-c^  (4.5) | 0.55^a-c^ | 1.71^a-c^  (209.5) | 0.28^e^ | 0.19^b^  (31.9) |
| LSD_(0.05)_ | 3.63 | 7.71 | 3.56 | 2.81 | 0.70 | 0.61 | 0.13 | 0.33 | 0.05 | 0.02 |
| CV (%) | 2.78 | 7.80 | 20.25 | 5.66 | 8.37 | 7.41 | 15.48 | 14.47 | 9.38 | 8.31 |

LSD, least significant difference; CV, coefficient of variations. Statistical analyses were conducted separately for drought and control treatments, with values derived from five biological replicates (*n* = 5; 10 plants per replicate). Distinct alphabetical symbols denote the order of significance (p < 0.05) for the traits analyzed, with a > b > c > d > e. Value in the parenthesis indicates the percent reduction and increased in drought stress compared to control. The parameters which have been marked with asterisk (*) that were increased under drought stress compared to control.

**Supplemental Table S5.** Biochemical attributes of investigated soybean genotypes under varying water regime conditions

| **Genotypes** | **Chlorophyll *a***  **(mg g^–1^ FW)** | | **Chlorophyll *b***  **(mg g^–1^ FW)** | | **Total chlorophylls**  **(mg g^–1^ FW)** | | **Carotenoids**  **(mg g^–1^ FW)** | | **Proline***  **(μg g^–1^ FW)** | | | **Malondialdehyde***  **(μmol g^–1^ FW)** | |
| --- | --- | --- | --- | --- | --- | --- | --- | --- | --- | --- | --- | --- | --- |
|  | **control** | **drought** | **control** | **drought** | **control** | **drought** | **control** | **drought** | **control** | **drought** | **control** | | **drought** |
| G00001 | 3.84^b^ | 3.05^a^  (20.7) | 2.12^a^ | 1.18^b^  (44.2) | 5.96^b^ | 4.23^a^  (29.0) | 8.35^bc^ | 7.34^a^  (12.2) | 2.39^b^ | 4.49^g^  (88.2) | 2.44^d^ | | 3.70^f^  (51.9) |
| G00046 | 3.39^cd^ | 2.26^b^  (33.4) | 1.64^b^ | 1.40^a^  (14.6) | 5.04^c^ | 3.67^b^  (27.2) | 7.84^cd^ | 6.38^b^  (18.6) | 2.22^b^ | 5.74^f^  (159.0) | 2.22^d^ | | 4.25^f^  (91.0) |
| G00135 | 3.20^d^ | 1.47^d^  (54.1) | 1.40^b^ | 1.07^bc^  (23.6) | 4.60^d^ | 2.54^d^  (44.9) | 9.17^a^ | 4.67^e^  (49.0) | 3.59^a^ | 10.22^b^  (184.4) | 4.05^b^ | | 12.63^b^  (211.9) |
| BD2333 | 4.98^a^ | 1.02^e^  (79.5) | 2.34^a^ | 0.98^cd^  (58.3) | 7.32^a^ | 2.00^e^  (72.7) | 8.77^ab^ | 3.80^f^  (56.7) | 3.70^a^ | 11.60^a^  (213.2) | 5.34^a^ | | 20.29^a^  (280.3) |
| PK472 | 2.40^e^ | 1.45^d^  (39.6) | 1.39^b^ | 1.11^bc^  (20.0) | 3.79^e^ | 2.56^d^  (32.4) | 8.42^bc^ | 5.11^cd^  (39.3) | 1.78^c^ | 6.61^e^  (271.7) | 3.97^b^ | | 7.02^e^  (77.1) |
| BARI Soybean6 | 3.65^bc^ | 2.14^bc^  (41.6) | 2.36^a^ | 0.85^d^  (64.2) | 5.97^b^ | 2.98^c^  (50.1) | 7.98^cd^ | 4.79^de^  (40.0) | 1.40^d^ | 8.86^c^  (532.3) | 3.47^c^ | | 9.59^c^  (176.2) |
| BU Soybean2 | 3.85^b^ | 1.93^c^  (49.8) | 2.33^a^ | 1.17^b^  (49.9) | 6.18^b^ | 3.10^c^  (49.8) | 7.44^d^ | 5.30^c^  (28.8) | 1.74^cd^ | 7.05^d^  (304.2) | 4.37^b^ | | 8.29^d^  (89.6) |
| LSD_(0.05)_ | 0.39 | 0.27 | 0.30 | 0.14 | 0.38 | 0.18 | 0.72 | 0.32 | 0.35 | 0.37 | 0.44 | | 0.97 |
| CV (%) | 7.27 | 9.46 | 10.28 | 8.79 | 4.62 | 4.09 | 5.85 | 4.12 | 9.94 | 3.20 | 8.03 | | 6.92 |

LSD, least significant difference, CV, coefficient of variations. Statistical analyses were conducted separately for drought and control treatments, with values derived from five biological replicates (*n* = 5; 10 plants per replicate). Value in the parenthesis indicates the percent reduction and increased under drought stress compared to control. Distinct alphabetical symbols denote the order of significance (p < 0.05) for the traits analyzed, with a > b > c > d > e > f. The parameters which have been marked with asterisk (*) that were increased under drought stress compared to control.

**Supplemental Table S6.** Correlation of studied traits of soybean genotypes under control (A) and drought stress (B) condition

A. Control

| **Traits** | **PH** | **LA** | **LP** | **BP** | **PP** | **SP** | **HSW** | **SY** | **RWC** | **WSD** | **WRC** | **WUC** | **XER** | ***Pn*** | ***gs*** | ***Ci*** | ***E*** | **LT** | **WUE**  **int** | **WUEins** | **Chl *a*** | **Chl *b*** | **Total Chls** | **Carote**  **noids** | **Proline** | **MDA** |
| --- | --- | --- | --- | --- | --- | --- | --- | --- | --- | --- | --- | --- | --- | --- | --- | --- | --- | --- | --- | --- | --- | --- | --- | --- | --- | --- |
| **PH** | 1 |  |  |  |  |  |  |  |  |  |  |  |  |  |  |  |  |  |  |  |  |  |  |  |  |  |
| **LA** | -0.78^*^ | 1 |  |  |  |  |  |  |  |  |  |  |  |  |  |  |  |  |  |  |  |  |  |  |  |  |
| **LP** | 0.68 | -0.24 | 1 |  |  |  |  |  |  |  |  |  |  |  |  |  |  |  |  |  |  |  |  |  |  |  |
| **BP** | 0.65 | -0.17 | .98^**^ | 1 |  |  |  |  |  |  |  |  |  |  |  |  |  |  |  |  |  |  |  |  |  |  |
| **PP** | 0.29 | -0.58 | -0.13 | -0.24 | 1 |  |  |  |  |  |  |  |  |  |  |  |  |  |  |  |  |  |  |  |  |  |
| **SP** | -0.11 | -0.14 | -0.69 | -0.68 | 0.51 | 1 |  |  |  |  |  |  |  |  |  |  |  |  |  |  |  |  |  |  |  |  |
| **HSW** | -0.42 | 0.27 | -0.39 | -0.31 | -0.13 | 0.43 | 1 |  |  |  |  |  |  |  |  |  |  |  |  |  |  |  |  |  |  |  |
| **SY** | 0.29 | -0.48 | -0.07 | -0.12 | 0.75 | 0.64 | 0.46 | 1 |  |  |  |  |  |  |  |  |  |  |  |  |  |  |  |  |  |  |
| **RWC** | -0.05 | 0.09 | 0.36 | 0.39 | -0.49 | -.87^**^ | -0.48 | -.78^*^ | 1 |  |  |  |  |  |  |  |  |  |  |  |  |  |  |  |  |  |
| **WSD** | -0.31 | 0.25 | -0.56 | -0.55 | 0.28 | .87^*^ | 0.66 | 0.60 | -.92^**^ | 1 |  |  |  |  |  |  |  |  |  |  |  |  |  |  |  |  |
| **WRC** | 0.22 | -0.24 | 0.29 | 0.34 | -0.28 | -0.64 | -0.62 | -0.65 | .88^**^ | -.89^**^ | 1 |  |  |  |  |  |  |  |  |  |  |  |  |  |  |  |
| **WUC** | -0.18 | 0.09 | -0.59 | -0.56 | 0.33 | .95^**^ | 0.57 | 0.61 | -.92^**^ | .96^**^ | -.76* | 1 |  |  |  |  |  |  |  |  |  |  |  |  |  |  |
| **XER** | 0.36 | 0.01 | 0.67 | 0.64 | 0.31 | -0.22 | -0.21 | 0.28 | -0.09 | -0.01 | -0.16 | -0.08 | 1 |  |  |  |  |  |  |  |  |  |  |  |  |  |
| ***Pn*** | 0.02 | -0.12 | -0.05 | -0.05 | 0.35 | 0.42 | .80^*^ | .85* | -0.65 | 0.61 | -0.73 | 0.52 | 0.21 | 1 |  |  |  |  |  |  |  |  |  |  |  |  |
| ***Gs*** | -0.24 | 0.25 | -0.08 | -0.04 | 0.06 | 0.32 | .88^**^ | 0.62 | -0.56 | 0.65 | -0.75 | 0.51 | 0.24 | .92^**^ | 1 |  |  |  |  |  |  |  |  |  |  |  |
| ***Ci*** | -0.04 | 0.24 | 0.19 | 0.17 | -0.20 | -0.48 | -.84^*^ | -0.75 | 0.60 | -0.56 | 0.61 | -0.52 | 0.15 | -.91^**^ | -.79^*^ | 1 |  |  |  |  |  |  |  |  |  |  |
| ***E*** | -0.10 | 0.06 | -0.07 | -0.04 | 0.16 | 0.39 | .88^**^ | 0.74 | -0.63 | 0.65 | -0.75 | 0.55 | 0.19 | .98^**^ | .98^**^ | -.90^**^ | 1 |  |  |  |  |  |  |  |  |  |
| **LT** | 0.27 | -0.22 | 0.21 | 0.23 | -0.16 | -0.47 | -.84^*^ | -0.67 | 0.71 | -.76^*^ | .90^**^ | -0.62 | -0.12 | -.91^**^ | -.94^**^ | .82^*^ | -.94^**^ | 1 |  |  |  |  |  |  |  |  |
| **WUEint** | 0.28 | -0.42 | -0.19 | -0.27 | 0.34 | 0.16 | -.76^*^ | -0.21 | 0.09 | -0.25 | 0.40 | -0.08 | -0.26 | -0.68 | -.86^*^ | 0.56 | -.78^*^ | 0.69 | 1 |  |  |  |  |  |  |  |
| **WUEins** | -0.03 | -0.14 | -0.20 | -0.28 | 0.25 | -0.09 | -.81^*^ | -0.43 | 0.36 | -0.40 | 0.52 | -0.31 | -0.17 | -.79^*^ | -.87^*^ | .78^*^ | -.88^**^ | .76^*^ | .88^**^ | 1 |  |  |  |  |  |  |
| **Chl *a*** | -0.38 | 0.49 | 0.18 | 0.32 | -0.52 | -0.56 | 0.15 | -0.46 | 0.64 | -0.36 | 0.43 | -0.42 | 0.16 | -0.12 | 0.13 | 0.22 | -0.01 | 0.15 | -0.53 | -0.20 | 1 |  |  |  |  |  |
| **Chl *b*** | -0.27 | 0.41 | 0.22 | 0.36 | -.79^*^ | -0.55 | 0.42 | -0.41 | 0.51 | -0.29 | 0.27 | -0.37 | -0.14 | 0.06 | 0.30 | -0.15 | 0.22 | -0.08 | -0.71 | -0.55 | .787* | 1 |  |  |  |  |
| **Total Chls** | -0.36 | 0.48 | 0.20 | 0.35 | -0.65 | -0.58 | 0.27 | -0.46 | 0.62 | -0.35 | 0.39 | -0.42 | 0.06 | -0.05 | 0.21 | 0.09 | 0.08 | 0.07 | -0.63 | -0.34 | .97^**^ | .91^**^ | 1 |  |  |  |
| **Carotenoids** | -0.27 | 0.41 | -0.23 | -0.23 | 0.03 | 0.06 | -0.57 | -0.46 | 0.10 | 0.01 | 0.16 | 0.05 | 0.13 | -0.69 | -0.54 | .82^*^ | -0.65 | 0.49 | 0.56 | 0.72 | 0.02 | -0.43 | -0.15 | 1 |  |  |
| **Proline** | -0.42 | 0.36 | -0.24 | -0.20 | 0.12 | -0.07 | -0.39 | -0.37 | 0.33 | -0.13 | 0.35 | -0.10 | 0.21 | -0.51 | -0.37 | 0.69 | -0.49 | 0.45 | 0.30 | 0.62 | 0.44 | -0.16 | 0.24 | .80^*^ | 1 |  |
| **MDA** | 0.06 | -0.10 | -0.03 | 0.074 | -0.38 | -0.34 | -0.48 | -0.68 | 0.70 | -0.62 | .89^**^ | -0.45 | -0.38 | -.78* | -0.75 | 0.59 | -0.75 | .87^*^ | 0.47 | 0.52 | 0.38 | 0.24 | 0.35 | 0.32 | 0.41 | 1 |

B. Drought stress

| **Traits** | **PH** | **LA** | **LP** | **BP** | **PP** | **SP** | **HSW** | **SY** | **RWC** | **WSD** | **WRC** | **WUC** | **XER** | ***Pn*** | ***gs*** | ***Ci*** | ***E*** | **LT** | **WUE**  **int** | **WUEins** | **Chl *a*** | **Chl *b*** | **Total Chls** | **Carote**  **noids** | **Proline** | **MDA** |
| --- | --- | --- | --- | --- | --- | --- | --- | --- | --- | --- | --- | --- | --- | --- | --- | --- | --- | --- | --- | --- | --- | --- | --- | --- | --- | --- |
| **LA** | 0.07 | 1.00 |  |  |  |  |  |  |  |  |  |  |  |  |  |  |  |  |  |  |  |  |  |  |  |  |
| **LP** | 0.21 | -0.07 | 1 |  |  |  |  |  |  |  |  |  |  |  |  |  |  |  |  |  |  |  |  |  |  |  |
| **BP** | 0.71 | -0.02 | 0.48 | 1 |  |  |  |  |  |  |  |  |  |  |  |  |  |  |  |  |  |  |  |  |  |  |
| **PP** | 0.59 | 0.33 | -0.29 | 0.41 | 1 |  |  |  |  |  |  |  |  |  |  |  |  |  |  |  |  |  |  |  |  |  |
| **SP** | 0.55 | 0.21 | -0.55 | 0.41 | .81^*^ | 1 |  |  |  |  |  |  |  |  |  |  |  |  |  |  |  |  |  |  |  |  |
| **HSW** | 0.56 | 0.47 | -0.04 | 0.69 | 0.68 | .79^*^ | 1 |  |  |  |  |  |  |  |  |  |  |  |  |  |  |  |  |  |  |  |
| **SY** | 0.73 | 0.55 | -0.09 | 0.52 | .87^*^ | .783^*^ | .851^*^ | 1 |  |  |  |  |  |  |  |  |  |  |  |  |  |  |  |  |  |  |
| **RWC** | 0.37 | 0.40 | 0.29 | 0.55 | 0.67 | 0.40 | 0.73 | 0.74 | 1 |  |  |  |  |  |  |  |  |  |  |  |  |  |  |  |  |  |
| **WSD** | -0.37 | -0.40 | -0.29 | -0.55 | -0.67 | -0.40 | -0.73 | -0.74 | -1.0^**^ | 1 |  |  |  |  |  |  |  |  |  |  |  |  |  |  |  |  |
| **WRC** | 0.03 | -0.15 | 0.64 | 0.02 | -0.75 | -0.70 | -0.45 | -0.48 | -0.48 | 0.48 | 1 |  |  |  |  |  |  |  |  |  |  |  |  |  |  |  |
| **WUC** | -0.30 | -0.38 | -0.10 | -0.47 | -0.75 | -0.52 | -0.74 | -0.75 | -.97^**^ | .97^**^ | 0.66 | 1 |  |  |  |  |  |  |  |  |  |  |  |  |  |  |
| **XER** | 0.63 | 0.60 | -0.21 | 0.17 | 0.37 | 0.51 | 0.47 | 0.66 | 0.06 | -0.06 | 0.06 | -0.02 | 1 |  |  |  |  |  |  |  |  |  |  |  |  |  |
| ***Pn*** | 0.58 | 0.48 | -0.15 | 0.48 | 0.69 | .811^*^ | .93^**^ | .91^**^ | 0.71 | -0.71 | -0.49 | -0.72 | 0.59 | 1 |  |  |  |  |  |  |  |  |  |  |  |  |
| ***Gs*** | 0.53 | 0.61 | -0.24 | 0.40 | 0.59 | .79^*^ | .90^**^ | .86^*^ | 0.54 | -0.54 | -0.39 | -0.56 | 0.73 | .96^**^ | 1 |  |  |  |  |  |  |  |  |  |  |  |
| ***Ci*** | -0.71 | -0.11 | 0.16 | -0.54 | -0.56 | -.82^*^ | -.80^*^ | -.76^*^ | -0.46 | 0.46 | 0.32 | 0.47 | -0.57 | -.89^**^ | -.84^*^ | 1 |  |  |  |  |  |  |  |  |  |  |
| ***E*** | 0.58 | 0.56 | -0.15 | 0.42 | 0.65 | .767^*^ | .88^**^ | .90^**^ | 0.66 | -0.66 | -0.41 | -0.66 | 0.68 | .99^**^ | .96^**^ | -.87^*^ | 1 |  |  |  |  |  |  |  |  |  |
| **LT** | -0.46 | -0.60 | 0.26 | -0.38 | -0.64 | -.81^*^ | -.92^**^ | -.86^*^ | -0.62 | 0.62 | 0.49 | 0.65 | -0.64 | -.97^**^ | -.99^**^ | .83^*^ | -.98^**^ | 1 |  |  |  |  |  |  |  |  |
| **WUEint** | -.76^*^ | -0.28 | -0.11 | -0.67 | -0.30 | -0.58 | -0.73 | -0.64 | -0.25 | 0.25 | -0.14 | 0.18 | -0.75 | -0.71 | -.78^*^ | .81^*^ | -0.73 | 0.70 | 1 |  |  |  |  |  |  |  |
| **WUEins** | -0.60 | -0.47 | -0.21 | -0.19 | -0.06 | -0.17 | -0.34 | -0.50 | -0.11 | 0.11 | -0.40 | -0.02 | -.85^*^ | -0.51 | -0.60 | 0.53 | -0.61 | 0.50 | 0.75 | 1 |  |  |  |  |  |  |
| **Chl *a*** | 0.27 | 0.58 | -0.07 | 0.47 | 0.49 | 0.65 | .93^**^ | 0.72 | 0.72 | -0.72 | -0.44 | -0.73 | 0.39 | .89^**^ | .89^**^ | -0.70 | .88^**^ | -.93^**^ | -0.61 | -0.34 | 1 |  |  |  |  |  |
| **Chl *b*** | 0.70 | 0.26 | -0.24 | 0.28 | .91^**^ | 0.72 | 0.55 | .87^*^ | 0.59 | -0.59 | -0.58 | -0.64 | 0.50 | 0.70 | 0.59 | -0.65 | 0.69 | -0.61 | -0.34 | -0.31 | 0.37 | 1 |  |  |  |  |
| **Total Chls** | 0.41 | 0.57 | -0.12 | 0.48 | 0.65 | 0.74 | .95^**^ | .85^*^ | .77^*^ | -.77^*^ | -0.53 | -.80^*^ | 0.46 | .96^**^ | .93^**^ | -.77^*^ | .94^**^ | -.97^**^ | -0.62 | -0.37 | .97^**^ | 0.57 | 1 |  |  |  |
| **Carotenoids** | 0.49 | 0.63 | -0.24 | 0.46 | .77^*^ | .83^*^ | .95^**^ | .91^**^ | 0.71 | -0.71 | -0.59 | -.75^*^ | 0.58 | .95^**^ | .95^**^ | -.76^*^ | .94^**^ | -.97^**^ | -0.63 | -0.38 | .91^**^ | 0.67 | .97^**^ | 1 |  |  |
| **Proline** | -0.70 | -0.40 | 0.21 | -0.64 | -0.74 | -.91^**^ | -.94^**^ | -.89^**^ | -0.56 | 0.56 | 0.44 | 0.59 | -0.65 | -.92^**^ | -.92^**^ | .88^**^ | -.89^**^ | .91^**^ | .815* | 0.44 | -.80^*^ | -0.66 | -.87^*^ | -.93^**^ | 1 |  |
| **MDA** | -0.62 | -0.24 | 0.14 | -0.74 | -.76^*^ | -.88^**^ | -.95^**^ | -.82^*^ | -0.65 | 0.65 | 0.54 | 0.69 | -0.39 | -.86^*^ | -.81^*^ | .83* | -.79^*^ | .825* | 0.70 | 0.19 | -.80^*^ | -0.60 | -.85^*^ | -.88^**^ | .95^*^ | 1 |

^*^Correlation is significant at the 0.05 level; ^**^Correlation is significant at the 0.01 level. PH, plant height; LA, leaf area plant^‒1^; LP, leaves number plant^‒1^; BP, branches number plant^‒1^; PP, pods number plant^‒1^; SP, seeds pod^‒1^; HSW, 100-seed weight; SY, seed yield plant^‒1^; RWC, relative water content; WSD, water saturation deficit; WRC, water retention capacity; WUC, water uptake capacity; XER, xylem exudation rate; *gs*, stomatal conductance; *Ci*, intercellular CO_2_ concentration; *E*, transpiration rate; LT, leaf temperature; WUEint, intrinsic water use efficiency and WUEins, instantaneous water use efficiency; *Pn*, photosynthesis rate; Chl *a*, chlorophyll a; Chl *b*, chlorophyll b; Total Chls, total chlorophylls; MDA, malondialdehyde.

**Supplemental Table S7.** Metrological conditions of the research site during the experiment period

| **Month** | **Air temperature (°C)** | | | **Humidity**  **(%)** | **Rainfall**  **(mm)** | **Evaporation**  **(mm)** |
| --- | --- | --- | --- | --- | --- | --- |
|  | **Maximum** | **Minimum** | **Average** |  |  |  |
| November 21 | 30.47 | 18.32 | 24.39 | 91.03 | 11.36 | 73.55 |
| December 21 | 26.55 | 14.81 | 20.68 | 88.32 | 69.97 | 54.27 |
| January 22 | 24.87 | 13.45 | 19.16 | 87.58 | 8.77 | 45.39 |
| February 22 | 26.11 | 12.84 | 19.47 | 87.36 | 30.68 | 57.75 |
| March 22 | 33.95 | 19.73 | 26.84 | 84.19 | 12.99 | 106.01 |
| April 22 | 34.67 | 26.25 | 30.46 | 84.07 | 55.52 | 138.23 |
| May 22 | 33.77 | 24.59 | 29.18 | 85.73 | 237.99 | 87.33 |
| Mean | 30.03 | 18.56 | 24.30 | 86.90 | 61.04 | 80.36 |

**Supplemental Table S8.** Name of the investigated agronomic traits, along with their acronyms, measurement units, and data collection procedures

| **Trait name** | **Acronym** | **Unit** | **Procedure** |
| --- | --- | --- | --- |
| Plant height | PH | cm | Shoot length was measured from the surface of the pot soil to the highest tip of the leaf |
| Leaves number plant^–1^ | LP | count | Visibility of the leaf petiole was considered as one leaf |
| Branches number plant^–1^ | BP | count | At least one leaf within the secondary stem was considered as one branch, and the number of branches per plant was counted. |
| Pods number plant^–1^ | PP | count | Fully mature pods in the plant was counted |
| Seeds pod^–1^ | SP | count | Fully developed seeds in the pods was counted |
| 100-seed weight | HSW | g | Weight of 100 grain was measured at 14% moisture content |
| Seed yield plant^–1^ | SY | g | Weight of grains per plant was measured at 14% moisture content |

**Supplemental Table S9.** Mathematical formulas of tolerance and susceptibility indices

| **Index** | **Formula** | **References** |
| --- | --- | --- |
| Tolerance | TOL = Y_P_ − Y_S_ | Rosielle and Hamblin [73] |
| Mean productivity | MP=(Y_P_+Y_S_)/2 | Rosielle and Hamblin [73] |
| Geometric mean productivity | GMP=√(Y_S_×Y_P_) | Fernandez [74] |
| Harmonic mean | HM=2(Y_S_×Y_P_)/(Y_S_+Y_P_) | Bidinger et al. [75] |
| Stress susceptibility index | SSI=1− (Y_S_/Y_P_)/1− (¯Ys/¯Yp) | Fischer and Maurer [76] |
| Stress tolerance index | STI=Y_S_×Y_P_/(¯Yp)^2^ | Fernandez [74] |
| Yield index | YI=Y_S_/¯Yp | Gavuzzi et al. [77] |
| Yield stability index | YSI=Y_S_/Y_P_ | Bouslama and Schapaugh [78] |
| Relative stress index | RSI=(Y_S_/Y_P_)/(¯Ys/¯Yp) | Fischer and Wood [79] |
